# Prognostic Value of Serum Neurofilament Light Chain for Disease Activity and Worsening in Patients With Relapsing Multiple Sclerosis: Results From the Phase 3 ASCLEPIOS I and II Trials

**DOI:** 10.3389/fimmu.2022.852563

**Published:** 2022-03-31

**Authors:** Tjalf Ziemssen, Douglas L. Arnold, Enrique Alvarez, Anne H. Cross, Roman Willi, Bingbing Li, Petra Kukkaro, Harald Kropshofer, Krishnan Ramanathan, Martin Merschhemke, Bernd Kieseier, Wendy Su, Dieter A. Häring, Stephen L. Hauser, Ludwig Kappos, Jens Kuhle

**Affiliations:** ^1^ Center of Clinical Neuroscience, Department of Neurology, University Clinic Carl-Gustav Carus, Dresden, Germany; ^2^ Brain Imaging Centre, Montreal Neurological Institute and Hospital, McGill University, Montreal, QC, Canada; ^3^ NeuroRx Research, Montreal, QC, Canada; ^4^ Department of Neurology, Rocky Mountain MS Center at the University of Colorado, Aurora, CO, United States; ^5^ Department of Neurology, Washington University School of Medicine, Saint Louis, MO, United States; ^6^ Novartis Pharma AG, Basel, Switzerland; ^7^ Novartis Pharmaceuticals Corporation, East Hanover, NJ, United States; ^8^ UCSF Weill Institute for Neurosciences, Department of Neurology, University of California, San Francisco, San Francisco, CA, United States; ^9^ Neurologic Clinic and Policlinic and MS Center, Department of Head, Spine and Neuromedicine, University Hospital Basel, Basel, Switzerland; ^10^ Research Center for Clinical Neuroimmunology and Neuroscience (RC2NB), Departments of Biomedicine and Clinical Research, University Hospital and University of Basel, Basel, Switzerland

**Keywords:** serum neurofilament light chain, prognostic biomarker, MS disease activity, lesion formation, brain atrophy

## Abstract

**Objective:**

This study aims to confirm the prognostic value of baseline serum neurofilament light chain (sNfL) for on-study disease activity and worsening in patients with relapsing MS (RMS).

**Background:**

Previous *post-hoc* studies suggested that sNfL could be a prognostic biomarker in RMS. In the phase 3 ASCLEPIOS I/II trials in which ofatumumab demonstrated better efficacy outcomes than teriflunomide, treatment with ofatumumab also led to significantly reduced sNfL levels compared to teriflunomide treatment.

**Design/Methods:**

In this study, we report protocol-planned analyses from the pooled ASCLEPIOS I/II trials (N=1882). Per protocol, patients were stratified by median baseline sNfL levels (9.3 pg/ml) into high (>median) and low (≤median) categories to prognosticate: annualized rate of new/enlarging T2 (neT2) lesions in year 1 and 2, annualized relapse rate, annual percentage change in whole brain (WB) and regional brain volume [thalamus, white matter (WM), cortical gray matter (cGM)], and disability outcomes. Similar analyses were performed for the recently diagnosed (within 3 years), treatment-naive patients (no prior disease-modifying therapy) subgroup.

**Results:**

High versus low sNfL at baseline was prognostic of increased on-study T2 lesion formation at year 1 (relative increase: ofatumumab +158%; teriflunomide +69%, both p<0.001), which persisted in year 2 (+65%, p=0.124; +46%, p=0.003); of higher annual percentage change of WB volume (ofatumumab, −0.32% vs. −0.24%, p=0.044, and teriflunomide, −0.43% vs. −0.29%, p=0.002), thalamic volume (−0.56% vs. −0.31%, p=0.047 and −0.94% vs. −0.49%, p<0.001), and WM volume (−0.30% vs. −0.19%, p=0.083 and −0.38% vs. −0.18%, p=0.003) but not of cGM volume (−0.39% vs. −0.32%, p=0.337 and −0.49% vs. −0.46%, p=0.563). A single sNfL assessment at baseline was not prognostic for on-study relapses or disability worsening. Results were similar in the subgroup of recently diagnosed, treatment-naive patients.

**Conclusion:**

This study confirms that baseline sNfL levels are prognostic of future on-study lesion formation and whole brain and regional atrophy in all RMS patients, including recently diagnosed, treatment-naive patients.

## 1 Introduction

Multiple sclerosis (MS) is a chronic, immune-mediated disease of the central nervous system (CNS), characterized by inflammation, demyelination, brain tissue, and neuronal loss, ultimately leading to severe disability (1). The hallmark of relapsing MS (RMS) are recurrent episodes of inflammatory demyelination in the brain and spinal cord, which can be accompanied by acute neurological symptoms (“MS relapse”), by nonspecific symptoms (e.g., pain or fatigue), or remain asymptomatic (i.e., subclinical MS lesion activity). The vast majority (~90%) of the focal inflammatory lesions in RMS, as measured on brain magnetic resonance imaging (MRI), are not associated with overt neurological symptoms but occur as subclinical lesion activity (2). However, despite most MRI lesions not being associated with acute neurological symptoms, lesion activities contribute to putatively irreversible changes such as neuronal damage and loss (3) and brain and spinal cord atrophy (4). MS-related changes in brain (5), thalamic (6, 7) and spinal cord volume (8), and neuronal injury/loss (9–11) have been shown to be associated with poor long-term outcomes in MS.

MS treatment guidelines recommend consideration of patients’ level of disease activity and severity when making the initial and subsequent treatment decisions (12, 13). However, there is currently no accepted consensus definition that allows physicians to classify RMS patients into either “high-risk” or “low-risk” groups in order to prioritize treatment strategies (12). A great challenge encountered in clinical practice concerning RMS patients is the difficulty in prognosticating the risk of future MS disease activity because of the extreme variability of MS disease course between patients (14) and the absence of prognostic tools. In current clinical practice, disease activity and severity are assessed mainly on the basis of the patient’s disease history and through evaluation of MRI, which are imperfect surrogates (15).

Biomarkers that can help to more reliably prognosticate future disease activity based on a standardized test could complement clinical and radiological assessments and would be of high value for decision making in routine clinical practice (3). Neurofilament light chain (NfL) is a specific biomarker of neuro-axonal injury released into the cerebrospinal fluid (CSF) and serum following neuro-axonal damage (16, 17). Serum and CSF NfL concentrations are highly correlated (18–23), and levels of serum NfL (sNfL) are higher in patients diagnosed with MS compared with age-matched healthy controls (3, 20, 24). NfL has been proposed as a prognostic and monitoring biomarker in MS to assess disease activity; the cumulative evidence for NfL has been reviewed elsewhere (25). In patients with RMS, high sNfL levels were found to correlate with active T2 lesions and relapses (3, 16, 20) and brain volume loss (26), suggesting that measuring sNfL levels can add value as a prognostic biomarker to identify patients at higher risk for future disease activity that can assist clinicians in decision-making (3, 18, 24, 27, 28).

There is currently an unmet need for a highly standardized, minimally invasive biomarker test, such as sNfL, to stratify diagnosed RMS patients into high- and low-risk groups based on the risk and intensity of anticipated future MS disease activity. The unmet need of prognosticating the risk of future MS disease activity is pronounced in newly diagnosed RMS patients who have not yet received a disease-modifying therapy (DMT) and in patients who discontinued from their prior treatment and aim to switch to another therapy. Several DMTs, including B-cell-depleting therapies, have shown an effect on lowering sNfL levels compared with untreated patients, independent of other variables (16, 29, 30). Initiating or switching to highly effective DMTs can decrease sNfL levels (3, 7, 16, 22) and improve long-term clinical outcomes for patients with RMS (31). Ofatumumab, a fully human anti-CD20 monoclonal antibody, selectively depletes B cells at a monthly dosing of 20 mg administered subcutaneously (30). In the phase 3 ASCLEPIOS I and II trials, ofatumumab significantly lowered sNfL levels at the first assessment at month 3 and at all subsequent visits compared with oral teriflunomide (14 mg daily) in patients with RMS (30).

The present analysis aims to test and confirm the prognostic value of baseline sNfL for clinical and sub-clinical disease activity and disability outcomes based on a pre-planned analysis in two large, well-controlled phase 3 trials (ASCLEPIOS I/II) in RMS. We hypothesized that patients with a “high” (>median) versus “low” (≤median) sNfL value at baseline would have higher number of demyelinating events as measured by new or enlarging T2 (neT2) lesions or clinical relapses and more brain volume loss.

## 2 Methods

### 2.1 Trial Design and Patients

This is a protocol-planned analysis from the pooled ASCLEPIOS I (NCT02792218) and ASCLEPIOS II (NCT02792231) trials (30) in patients with RMS who were randomly assigned (1:1) to receive either subcutaneous ofatumumab 20 mg every 4 weeks (starting at week 4, after initial doses of 20 mg on days 1, 7, and 14) or oral teriflunomide 14 mg once daily, for up to 30 months. Briefly, ASCLEPIOS I and II were randomized, double-blind, double-dummy, active-controlled, multicenter trials of identical design conducted concurrently in patients with RMS. Patients aged 18–55 years; a diagnosis of relapsing MS (either relapsing–remitting MS or secondary progressive MS with disease activity); an Expanded Disability Status Scale (EDSS) score of 0–5.5 (inclusive); at least one relapse in the year before screening or at least two relapses in the 2 years before screening or at least one gadolinium-enhancing (Gd+) lesion on MRI in the year before randomization; and a neurologically stable condition for at least a month before randomization were included in the study (30). Protocol-required washout periods for patients who transitioned off another DMT (i.e., treatment switchers) are detailed in Hauser et al. (30). The protocols were approved by the relevant institutional review board or ethics committee at each trial site, and all patients provided written informed consent.

### 2.2 NfL Assay and Performance Characteristics

Quantitation of NfL in human serum was performed centrally at one site, Navigate BioPharma Services, Carlsbad, CA, using the Quanterix Simoa NF-light assay advantage kit, which is a two-step quantitative digital immunoassay. Samples were collected from patients between the start and end of the study, i.e., between August 2016 and July 2019. Samples were analyzed in a single batch at the end of the study; maximal storage time was <3 years. For pre-processing of samples, approximately 3.5ml of blood was collected to serum separator tubes. Samples were inverted for a minimum of five times, and samples were allowed to clot 30–60 min at room temperature before centrifugation with a swing bucket for 10 min at 1,100 to 1,500 G-force or 15 min in a fixed angle centrifuge. Serum was aliquoted to pre-chilled cryovials and frozen at −70°C. Samples were shipped on dry ice and stored at a central laboratory until further testing.

Prior to the analysis of study samples, a technical assessment was performed to validate the performance claims of the Quanterix Simoa NfL kit in a serum matrix for use as a clinical trial assay. Lower limit of blank, lower limit of detection, and lower limit of quantification (LLoQ) were first established according to Clinical and Laboratory Standards Institute (CLSI) guideline (0.406, 2.05, and 2.05 pg/ml, respectively) (32). The LLoQ and upper limit of quantification (ULoQ) for the assay were verified by testing two QC samples, prepared at a nominal concentration of 2.05 and 1,900 pg/ml, in 60 measurements each, in duplicate. The LLoQ and ULoQ were defined as the means of the 60 measurements, with total precision (%CV) within the predefined acceptance criteria (≤25% at LLoQ, ≤20% at ULoQ). The LLoQ and ULoQ were verified at 2.8 and 1,546 pg/ml, establishing the reportable range. Intra-assay precision was demonstrated by testing eight samples in duplicate, across the assay reportable range independently six times in a single run, with the highest observed coefficient of variation (CV) of 10%. Interassay precision was demonstrated by testing eight samples in duplicate, independently three times per run for six runs (two runs per day), with the highest observed CV of 11%.

### 2.3 Study Outcomes and Assessments

All study objective, outcomes, and assessments were prospectively defined in the study protocols of ASCLEPIOS I and II. Baseline sNfL concentration was assessed prior to first dose of study treatment (ofatumumab or teriflunomide). MRI scans were performed at baseline, at months 12 and 24, and end-of-treatment/end-of-study (EOT/EOS, if no other scan was available within 3 months).

The prognostic value of baseline sNfL for on-study disease activity and worsening was analyzed for the annualized rate of neT2 lesion formation, annualized relapse rate (ARR), and annualized rate of percentage whole brain volume change and percentage volume change in regional brain compartments: thalamic volume, hemispheric white matter (WM), and cortical gray matter (cGM). To investigate the prognostic value of sNfL on future disease activity and worsening in patients who appeared “free of disease activity” at baseline based on the radiological assessment, similar analyses of lesion formation and brain volume loss were performed in the subset of patients without Gd+ T1 lesions at baseline. The time to first 3- and 6-month confirmed disability worsening (3m/6mCDW) were summarized descriptively, as the studies were not powered for this assessment, as acknowledged in the study protocols. To assess the clinical utility of sNfL as a prognostic tool in early RMS patients, similar analyses were performed in the protocol-defined subgroup of patients who were recently diagnosed (i.e., had the diagnosis of MS within 3 years) and who were treatment naive at baseline (i.e., had not received a prior MS treatment).

### 2.4 Statistical Methods

The statistical methodology, including NfL thresholds and subgroup definitions, was predefined in the statistical analysis plan of ASCLEPIOS I and II in year 2016, before any study data were collected. The pooled ASCLEPIOS I and II trial data were carried out on the full analysis set that comprised all randomized patients with assigned treatments. The baseline sNfL cutoff was predefined by the median NfL value across both studies to stratify a typical RMS population (as per the inclusion and exclusion criteria of the ASCLEPIOS I and II trials) into “high” and “low” sNfL groups of similar sample size before collecting any data. After completion of the phase 3 studies, the median baseline sNfL level was determined as 9.3 pg/ml in the statistical analysis, and patients were stratified accordingly into high (>9.3 pg/ml) and low (≤9.3 pg/ml) sNfL categories. The prognostic effect of baseline sNfL on MS disease activity (i.e., inflammatory demyelinating events) was performed based on MRI lesions or clinical relapses. The annualized rate of neT2 lesions was analyzed using a negative binomial regression model adjusted for treatment, baseline sNfL category, region, and study as factors; age and baseline volume of T2 lesions as continuous covariates; and treatment by baseline sNfL category interaction and the natural log of the time from the baseline scan (in years) as an offset. ARR was analyzed using a negative binomial regression model with log-link to the number of relapses, adjusted for treatment, baseline sNfL category, region, and study as factors; number of relapses in the previous year, baseline EDSS score, baseline number of Gd+ T1 lesions, and patient’s age at baseline as covariates; and treatment by baseline NfL category interaction and the natural log of the time-in-study as an offset.

The prognostic effect of baseline sNfL on MS disease worsening was assessed based on brain volume change and a descriptive analysis of disability worsening. The annual rate of both whole and regional brain volume percent changes was analyzed using the random coefficients model adjusted for study, treatment, region, and baseline sNfL category as fixed effects factors; time, baseline number of Gd+ T1 lesions, baseline T2 volume, and normalized brain volume (whole or regional) at baseline as continuous covariates; and treatment-by-time-by-baseline sNfL category interaction and the three corresponding two-way interactions. Random terms for slopes and the intercept were also included. The annual rate of brain volume loss was estimated based on the slope from a random coefficient model in the post-baseline assessments, i.e., the slope is estimated between year 1 and 2. Pearson correlation coefficients between baseline sNfL and regional brain volume changes at months 12 and 24 were computed. Disability worsening was described based on a Cox regression model adjusted for study as stratum, treatment, region, and baseline sNfL category as factors and baseline EDSS score as a continuous covariate along with treatment-by-baseline sNfL category interaction. The statistical hypothesis test of the prognostic value of sNfL for disability changes was based on the main effect of sNfL category (due to the expected low number of events in this subgroup). It is acknowledged that the study was not powered to show an effect on disability outcomes in subgroups.

## 3 Results

### 3.1 Patients

Of the 1,882 patients in the ASCLEPIOS I and II trials, 1,746 (92.8%) patients had serum NfL samples available at baseline. Of these, 870 (49.8%) were categorized as “high” and 876 (50.2%) as “low” baseline sNfL group. The baseline demographic and disease characteristics of patients by “high” and “low” sNfL are summarized in **Table 1**. The mean age, disease duration, and number of relapses in the previous year before the study were similar between the groups. The median baseline sNfL level was 6.76 pg/ml in the low and 14.23 pg/ml in the high sNfL categories. Patients with high sNfL levels differed from those with low sNfL levels by having a higher baseline T2 lesion volume (16.7 vs. 9.4 cm^3^), higher mean number of Gd+ T1 lesions (2.6 vs. 0.4), and a lower probability of being free of Gd+ T1 lesions (44% vs. 78%). Of the 870 high sNfL patients, 455 (52.3%) were randomly allocated to ofatumumab and 415 (47.7%) to teriflunomide; and of the 876 low sNfL patients, 438 (50.0%) were randomly allocated to ofatumumab and 438 (50.0%) to teriflunomide.

**Table 1:** Demographics and baseline characteristics in the overall study population, and in the subgroup of recently diagnosed, treatment-naive patients, by sNfL baseline high–low.

	All patients		Recently diagnosed, treatment-naive patients	
Parameters	Low sNfL category (≤9.3 pg/ml)	High sNfL category (>9.3 pg/ml)	Low sNfL category (≤9.3 pg/ml)	High sNfL category (>9.3 pg/ml)
	N=876*	N=870*	N=274*	N=302*
Age (years)	38.6 ± 8.5	37.8 ± 9.7	36.7 ± 8.8	35.9 ± 9.7
Sex, female, n (%)	588 (67.1)	602 (69.2)	180 (65.7)	209 (69.2)
MS duration since first symptom (years)	8.3 ± 7.3	7.9 ± 6.9	3.5 ± 4.4	3.1 ± 3.6
Previously treated with DMT	560 (59.2)	573 (61.2)	–	–
Number of relapses in the year before the study	1.2 ± 0.7	1.3 ± 0.7	1.3 ± 0.7	1.3 ± 0.7
Time since onset of most recent relapse (months)	7.8 ± 13.5	7.0 ± 9.4	5.8 ± 4.8	5.8 ± 5.7
EDSS score	2.8 ± 1.3	3.0 ± 1.4	2.2 ± 1.2	2.3 ± 1.2
Normalized brain volume (cm^3^)	1447.6 ± 74.8	1437.2 ± 81.0	1478.4 ± 64.9	1468.2 ± 71.1
Number of Gd+ T1 lesions	0.4 ± 1.2	2.6 ± 5.4	0.4 ± 1.0	2.6 ± 4.8
Patients free of Gd+ T1 lesions, n (%)	679 (77.5)	383 (44.0)	206 (75)	116 (38)
T2 lesion volume (cm^3^)	9.4 ± 10.6	16.7 ± 15.1	5.9 ± 7.2	12.3 ± 12.4
sNfL (pg/ml), median	6.76	14.23	6.77	15.29

The baseline sNfL level used for high–low stratification was assessed prior to first study treatment. Data are expressed as mean ± SD unless specified otherwise.

*Only patients with non-missing baseline NfL values are included.

EDSS, expanded disability status scale; Gd+, gadolinium-enhancing; SD, standard deviation; sNfL, serum neurofilament light chain.

The baseline characteristics of the subgroup of recently diagnosed treatment-naive patients (n=576) are presented in (**Table 1**). Recently diagnosed, treatment-naive patients had a shorter disease duration, were less disabled, and had a higher brain volume and a lower T2 lesion volume compared with the overall population. The median baseline sNfL level was 6.77 pg/ml in the low and 15.29 pg/ml in the high sNfL category. As expected, patients with high sNfL compared to those with low sNfL levels at baseline showed higher baseline T2 lesion volume (12.3 vs. 5.9 cm^3^), higher mean number of Gd+ T1 lesions (2.6 vs. 0.4), and a lower probability of being free of Gd+ T1 lesions (38% vs. 75%).

### 3.2 Prognostic Value of sNfL

#### 3.2.1 All Patients

##### 3.2.1.1 Disease Activity as Measured by MRI Lesions or Clinical Relapses

**Figure 1:**
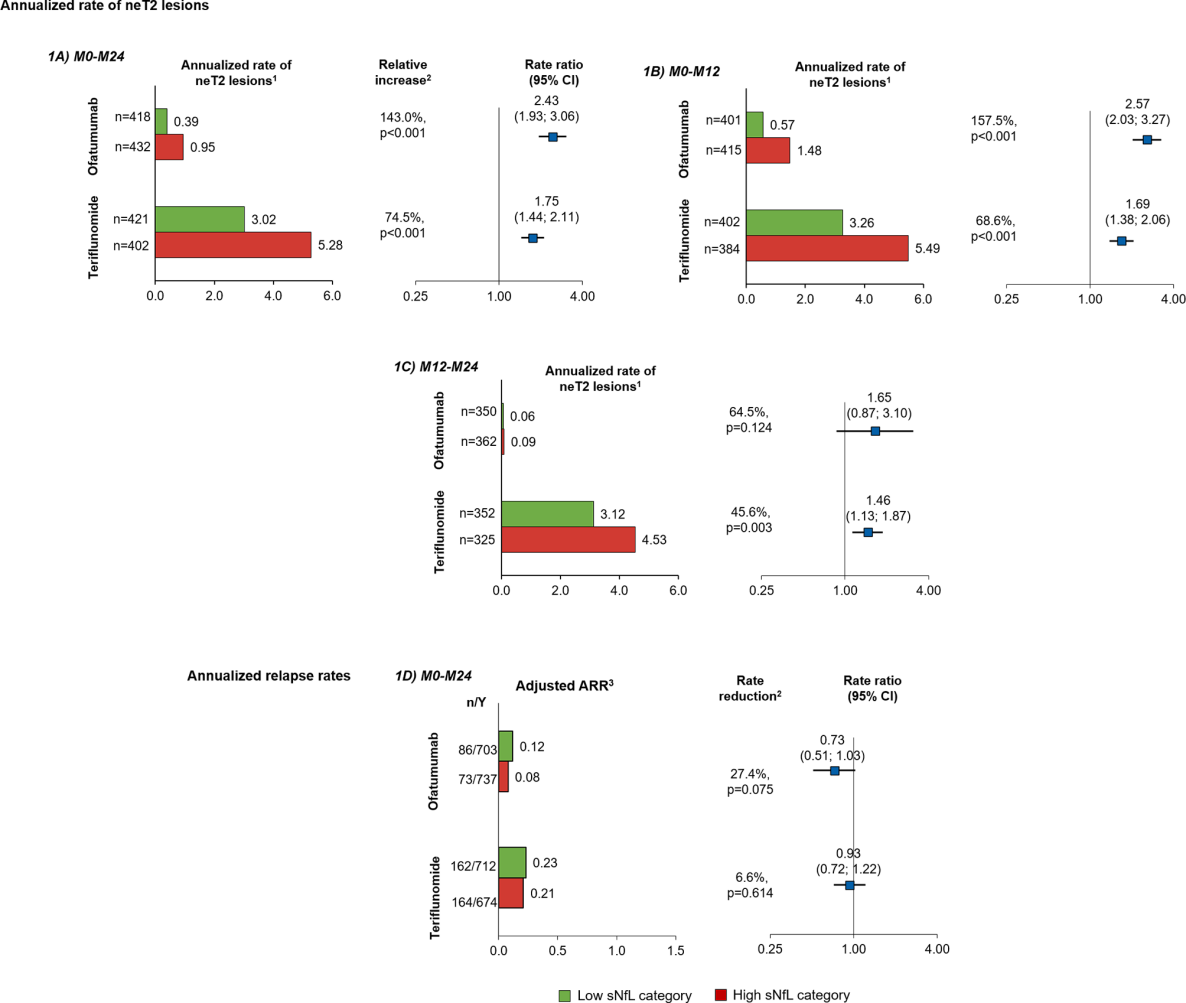
Prognostic value of baseline sNfL [high (red), ≥9.3 pg/ml; low (green), <9.3 pg/ml] on measures of disease activity in the overall study population: annualized rate of neT2 lesions per year by baseline sNfL category in the interval **(A)** M0–M24, **(B)** M0–M12, and **(C)** M12–M24; **(D)** ARR M0–M24. Comparisons are between high vs. low sNfL categories. ^1^Adjusted annualized mean rate of neT2 lesions. The number of neT2 lesions (compared to baseline or month 12) was analyzed in a negative binomial model with adjustments for treatment, baseline sNfL category, region and study as factors, and age and baseline volume of T2 lesions as continuous covariates, and treatment by baseline sNfL category interaction. The natural log of the time from the baseline scan (in years) was used as the offset. ^2^Indicates statistical significance (two-sided) at the 0.05 level. ^3^The ARR was analyzed in a negative binomial regression model with log-link to the number of relapses adjusted for treatment, baseline sNfL category, region and study as factors, number of relapses in previous year, baseline EDSS, baseline number of Gd+T1 lesions, and the patient’s age at baseline as covariates and treatment by baseline sNfL category interaction. The natural log of the time in study was used as offset to annualize the relapse rate. ARR, annualized relapse rate; CI, confidence interval; Gd+ gadolinium-enhancing; n, total number of relapses included in the analysis; neT2, new or enlarging T2 lesions; sNfL, serum neurofilament light; Y, number of patient-years in study.

The prognostic value of baseline sNfL for on-study disease activity was assessed based on MRI lesion formation (number of neT2 lesions) and clinical relapses (ARR). Within each treatment arm, patients with high sNfL levels at baseline showed higher on-study accumulation of neT2 lesions per year; neT2 lesions were 2.4 times in the ofatumumab group (143% increase, p<0.001) and 1.75 times higher (75% increase, p<0.001) in the teriflunomide group compared to those with low sNfL levels at baseline (**Figure 1A**). High versus low sNfL at baseline was prognostic of increased neT2 lesions both at year 1 (relative increase: ofatumumab, 157.5% and teriflunomide, 68.6%, both p<0.001; **Figure 1B**) and year 2 (relative increase: ofatumumab, 64.5%, p=0.124 and teriflunomide, 45.6%, p=0.003; **Figure 1C**). Even in the subgroup of patients free of Gd+ T1 lesions at baseline, i.e., those who would appear free of disease activity based on the radiological assessment, high compared with low baseline sNfL was still prognostic of significantly higher rate of annualized T2 lesion formation in both ofatumumab and teriflunomide groups (**Supplementary Figures 1A, B**).

In these active-controlled clinical trials, sNfL at baseline was not prognostic for on-study ARR, and relapse rates were not statistically different between high and low sNfL groups (relative reduction: ofatumumab, 27.0%, p=0.075; teriflunomide, 6.6%, p=0.614; **Figure 1D**). However, ARR was not high in either treatment group: pooled across the two trials, the ARR was 0.11 in the ofatumumab arm and 0.24 in the teriflunomide arm, which corresponds to one clinical relapse within 9 years in ofatumumab treated patients and one clinical relapse within 4 years in the teriflunomide arm.

##### 3.2.1.2 Disease Worsening as Measured by Brain Volume Change and Disability Outcomes

**Figure 2:**
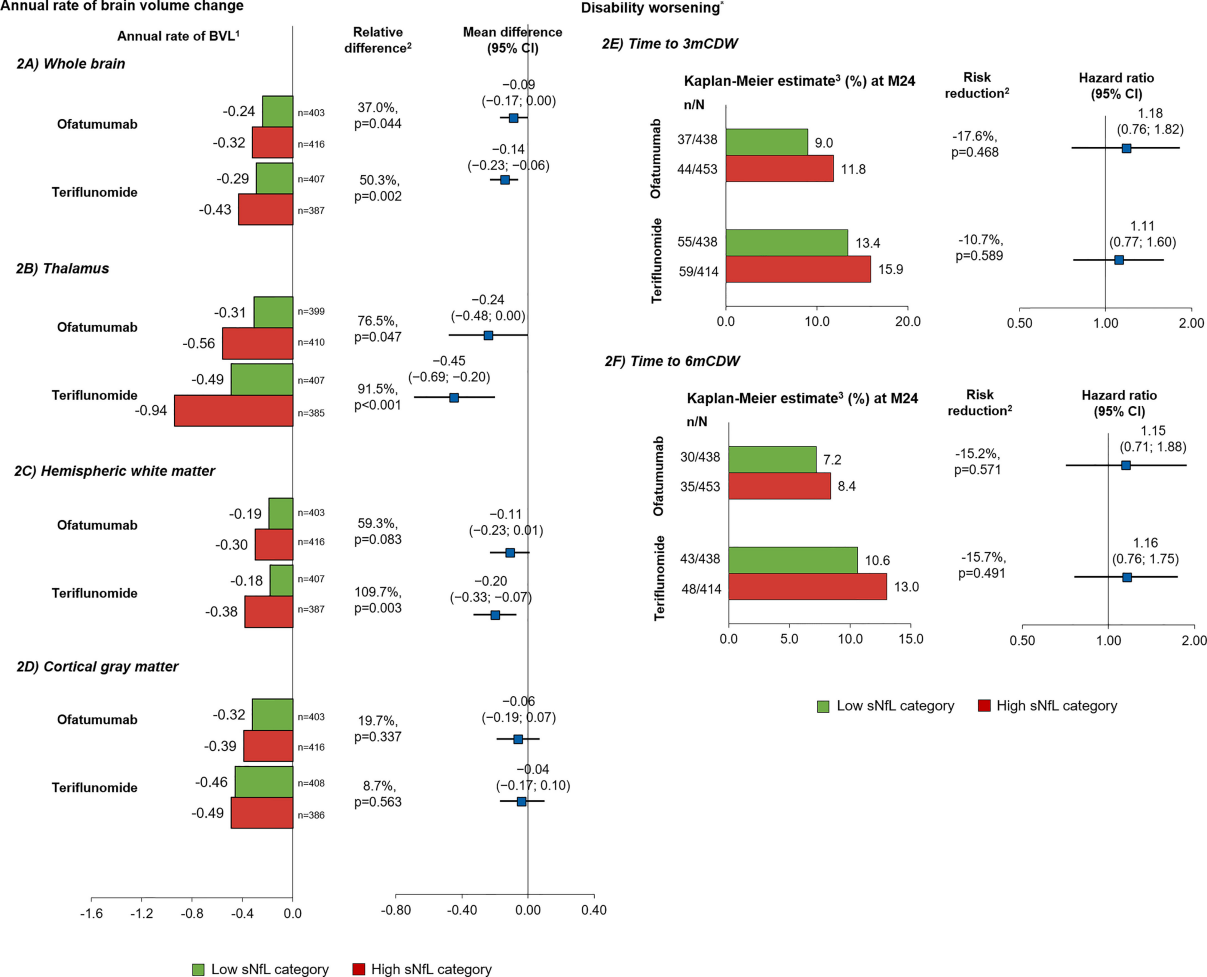
Prognostic value of baseline sNfL [high (red), ≥9.3 pg/ml; low (green), <9.3 pg/ml] on brain volume change and disability worsening in the overall study population*. Annual rate of brain volume change in **(A)** whole brain, **(B)** thalamus, **(C)** hemispheric white matter, and **(D)** cGM per year; time to disability worsening **(E)** 3mCDW and **(F)** 6mCDW by NfL baseline category. Comparisons are between high vs. low sNfL categories. ^1^Adjusted annual rate of brain volume change as estimated in from the random coefficient model with study fit to the post-baseline assessments measured at M12, M24, and end-of-study, treatment as fixed effects (factors), and time as continuous covariates and treatment by time interaction for the overall analysis, with additional co-factors of subgroup, treatment by subgroup, treatment by time by subgroup interactions for the subgroup analysis. Random terms for slopes and intercept are included. ^2^Indicates statistical significance (two-sided) at the 0.05 level. Obtained from a random coefficients model. ^3^Cox regression adjusted study as stratum, treatment, region, and sNfL baseline high–low subgroup as factors and baseline EDSS as a continuous covariate along with treatment-by-sNfL baseline high–low subgroup interaction. ^*^Descriptive analysis; the study was not powered to analyze prognostic effects of sNfL on disability outcomes. 3m, 3month; 6m, 6month; ARR, annualized relapse rate; CDW, confirmed disability worsening; cGM, cortical gray matter; CI, confidence interval; EDSS, Expanded Disability Status Scale Score; Gd+, gadolinium-enhancing; LS, least square; M, month; sNfL, serum neurofilament light; WM, white matter.

Within each treatment arm, patients with high sNfL levels at baseline lost significantly more whole brain volume per year (slope between year 1 and 2) than those with low sNfL levels (relative difference: ofatumumab, 37.0%, p=0.044; teriflunomide, 50.3%, p=0.002; **Figure 2A**). For the subgroup of patients with and without Gd+ T1 lesions at baseline, high compared with low baseline sNfL was associated with statistically significantly higher brain volume loss in the teriflunomide group, with a similar but non-significant trend also noted in the ofatumumab group (**Supplementary Figures 2A, B**).

**Figure 3:**
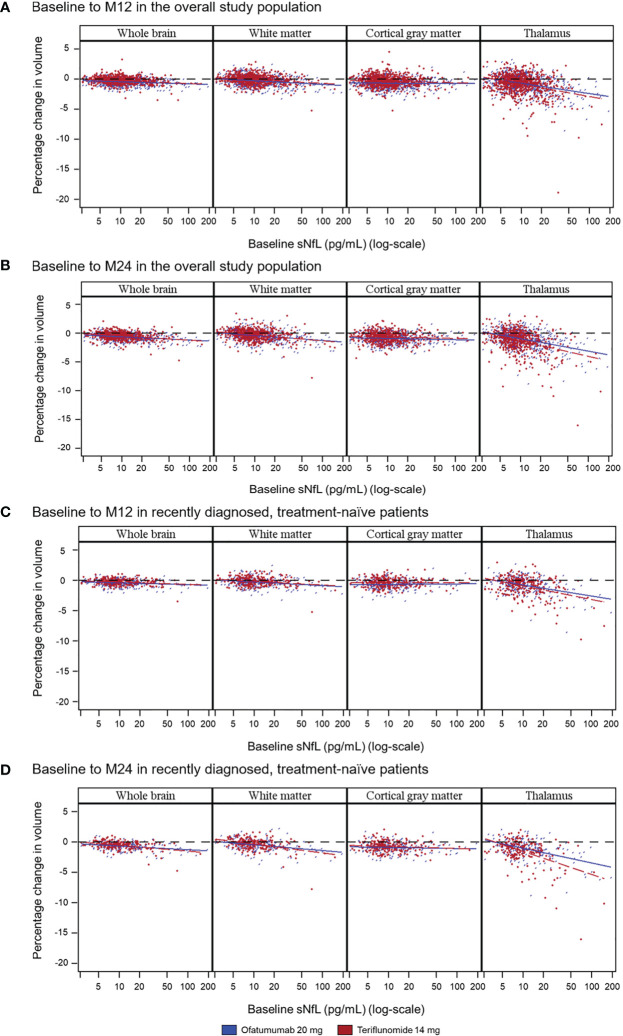
Correlation between baseline sNfL concentrations and on-study whole and regional brain volume change as illustrated in scatter plots over the specified time period in either the overall population (**A**: baseline to M12; **B**: baseline to M24), or in the subgroup of recently diagnosed, treatment-naive patients (**C**: baseline to M12; **D**: baseline to M24). Random coefficients model adjusted for study, treatment, region, and baseline sNfL category as fixed effects factors; time, baseline number of Gd+ T1 lesions, baseline T2 volume, and normalized brain volume (whole or regional) at baseline as continuous covariates; and treatment-by-time-by-baseline sNfL category interaction and the three corresponding two-way interactions. Gd+, gadolinium-enhancing; M, month; NfL, neurofilament light chain.

The difference in brain volume between patients with a high (vs. low) baseline NfL level was most pronounced in the thalamus (relative difference: ofatumumab, 76.5%, p=0.047; teriflunomide, 91.5%, p<0.001) (**Figure 2B**) and also was prognostic of a higher annual volume change between year 1 and 2 in hemispheric WM (59.3%, p=0.083; 109.7%, p=0.003) (**Figure 2C**) but not of cGM volume change (19.7%, p=0.337; 8.7%, p=0.563) (**Figure 2D**). Baseline sNfL correlated significantly with the percentage change from baseline to month 12 in whole brain volume (ofatumumab r=−0.200; teriflunomide r=−0.203, both p<0.0001), thalamic volume change (−0.374; −0.308, both p<0.0001), and WM (−0.230; −0.207, both p<0.0001) but less so with cGM volume change (−0.026, p=0.4679; −0.070, p=0.0523). Similar results were observed for the percentage change from baseline to month 24. The strongest correlation of sNfL change was with thalamic volume loss, both over 1 and 2 years (**Table 2**; **Figures 3A, B**).

**Table 2:** Pearson correlation coefficient between log-transformed baseline sNfL and percentage volume change in different brain compartments at months 12 and 24 from baseline in the overall study population and subgroup of recently diagnosed and treatment-naive patients.

Compartment	Treatment	*All patients*				*Recently diagnosed, treatment-naive patients*			
		N	Correlation	95% CI	*p-value*	N	Correlation	95% CI	*p-value*
*Baseline to month 12*									
Whole brain	Ofatumumab 20 mg	795	**−0.200**	(−0.266, −0.132)	*<0.0001*	256	**−0.199**	(−0.314, −0.078)	*0.0013*
	Teriflunomide 14 mg	760	**−0.203**	(−0.270, −0.134)	*<0.0001*	272	**−0.172**	(−0.285, −0.054)	*0.0044*
Thalamus	Ofatumumab 20 mg	785	**−0.374**	(−0.432, −0.312)	*<0.0001*	255	**−0.379**	(−0.480, −0.269)	*<0.0001*
	Teriflunomide 14 mg	758	**−0.308**	(−0.371, −0.242)	*<0.0001*	265	**−0.399**	(−0.495, −0.292)	*<0.0001*
Hemispheric white matter	Ofatumumab 20 mg	794	**−0.230**	(−0.295, −0.163)	*<0.0001*	256	**−0.234**	(−0.347, −0.115)	*0.0001*
	Teriflunomide 14 mg	760	**−0.207**	(−0.275, −0.138)	*<0.0001*	272	**−0.225**	(−0.335, −0.109)	*0.0002*
Cortical gray matter	Ofatumumab 20 mg	795	−0.026	(−0.095, 0.044)	*0.4679*	257	−0.006	(−0.128, 0.117)	*0.9289*
	Teriflunomide 14 mg	760	−0.070	(−0.141, 0.001)	*0.0523*	271	0.042	(−0.078, 0.160)	*0.4958*
*Baseline to month 24*									
Whole brain	Ofatumumab 20 mg	624	**−0.282**	(−0.353, −0.209)	*<0.0001*	213	**−0.384**	(−0.493, −0.263)	*<0.0001*
	Teriflunomide 14 mg	597	**−0.269**	(−0.342, −0.193)	*<0.0001*	203	**−0.308**	(−0.427, −0.177)	*<0.0001*
Thalamus	Ofatumumab 20 mg	614	**−0.416**	(−0.479, −0.348)	*<0.0001*	208	**−0.473**	(−0.572, −0.360)	*<0.0001*
	Teriflunomide 14 mg	588	**−0.367**	(−0.435, −0.295)	*<0.0001*	195	**−0.460**	(−0.564, −0.342)	*<0.0001*
Hemispheric white matter	Ofatumumab 20 mg	623	**−0.292**	(−0.362, −0.218)	*<0.0001*	213	**−0.409**	(−0.515, −0.290)	*<0.0001*
	Teriflunomide 14mg	597	**−0.286**	(−0.358, −0.210)	*<0.0001*	202	**−0.311**	(−0.430, −0.181)	*<0.0001*
Cortical gray matter	Ofatumumab 20 mg	624	−0.080	(−0.157, −0.001)	*0.0466*	214	−0.095	(−0.226, 0.040)	*0.1674*
	Teriflunomide 14mg	598	−0.068	(−0.147, 0.012)	*0.0965*	203	−0.075	(−0.210, 0.063)	*0.2883*

N, number of patients included in the analysis. Negative correlation coefficients signify the high sNfL values were correlated with negative brain volume values. Significant correlation coefficients are presented in bold.

CI, confidence interval; sNfL, serum neurofilament light chain.

The difference in the risk of 3mCDW (**Figure 2E**) or 6mCDW (**Figure 2F**) was not statistically significant between high and low sNfL groups, but trended in the expected direction, consistent with the change in brain volume.

#### 3.2.2 Subgroup of Recently Diagnosed and Treatment-Naive Patients

##### 3.2.2.1 Risk of Future Demyelinating Events as Measured by the Number of T2 Lesion Formation and Relapses

**Figure 4:**
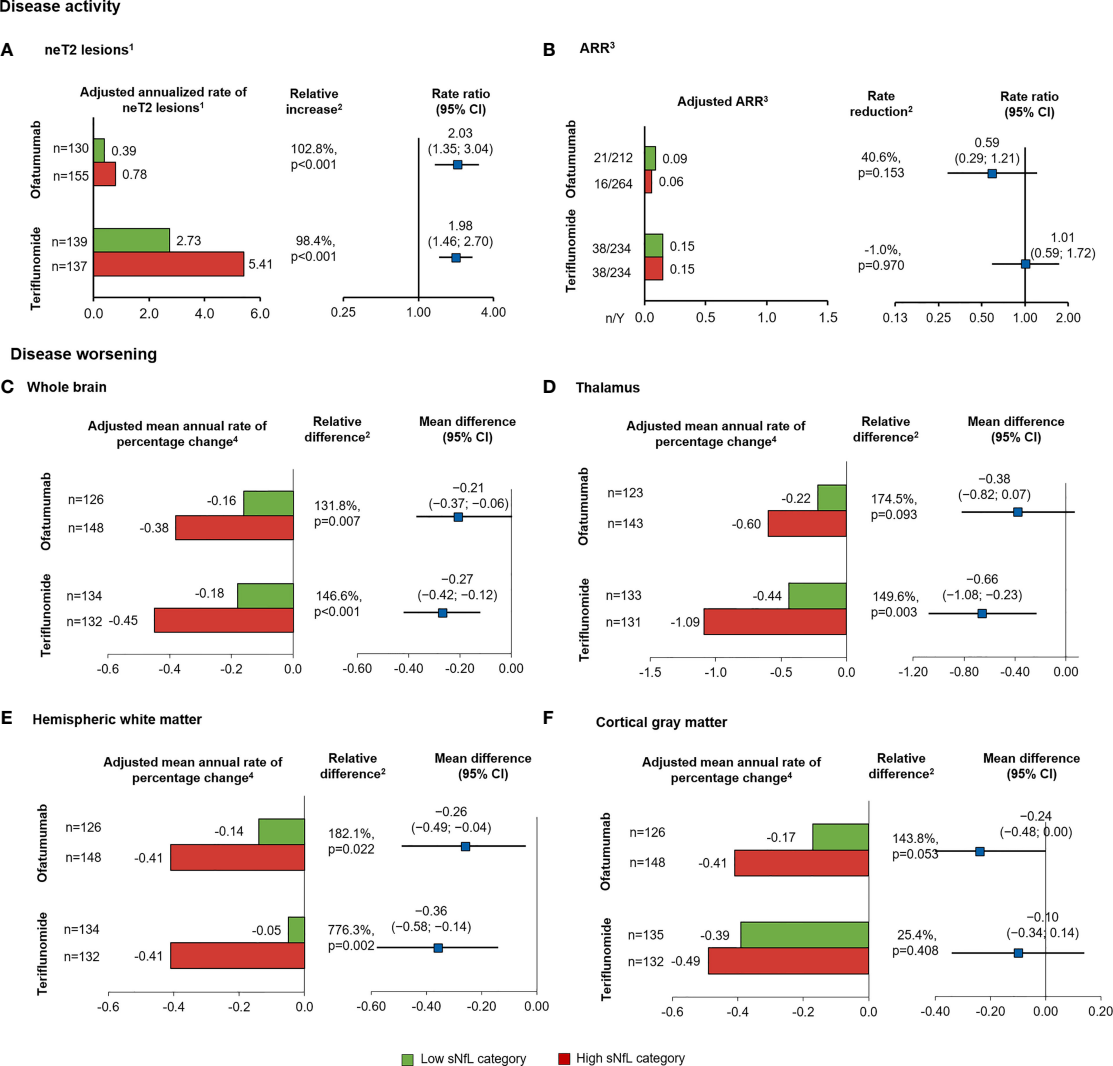
Prognostic value of baseline sNfL category [high (red), ≥9.3 pg/ml; low (green), <9.3 pg/ml) on measures of MS disease activity and worsening, in the subgroup of recently diagnosed, treatment-naive patients. **(A)** neT2 lesions per year, **(B)** annualized relapse rate. Annual rate of brain volume change in the **(C)** whole brain, **(D)** thalamic volume, **(E)** hemispheric WM, **(F)** cortical gray matter. Comparisons are between high vs. low sNfL categories. ^1^The number of neT2 lesions (compared to baseline or month 12) was analyzed in a negative binomial model with adjustments for treatment, baseline sNfL category, region and study as factors, and age, baseline volume of T2 lesions as continuous covariates, and treatment by baseline sNfL category interaction. The natural log of the time from the baseline scan (in years) was used as the offset. ^2^Indicates statistical significance (two-sided) at the 0.05 level. ^3^Negative binomial regression model with log-link to the number of relapses adjusted for treatment, baseline sNfL category, region and study as factors, number of relapses in previous year, baseline EDSS, baseline number of Gd+T1 lesions, and the patient’s age at baseline as covariates and treatment by baseline sNfL category interaction. The natural log of the time in study was used as offset to annualize the relapse rate. ^4^Annual rate of brain volume change obtained from a random coefficients model with study, treatment as fixed effects (factors), and time as continuous covariates and treatment by time interaction for the overall analysis, with additional co-factors of subgroup, treatment by subgroup, and treatment by time by subgroup interactions for the subgroup analysis. Random terms for slopes and intercept are included. ARR, annualized relapse rate; BVL, brain volume loss; CI, confidence interval; cGM, cortical gray matter; EDSS, Expanded Disability Status Scale score; Gd+, gadolinium-enhancing; neT2, new or enlarging T2 lesions; LS, least square; sNfL, serum neurofilament light; WM, white matter.

To assess the prognostic value of sNfL in patients who were recently diagnosed and treatment naive, a subgroup analysis was conducted. Patients with high compared with low sNfL levels at baseline developed more neT2 lesions per year during the study (relative increase: ofatumumab, 102.8%, p<0.001; teriflunomide, 98.4%, p<0.001; **Figure 4A**). However, the ARR was not statistically different between the groups and was low in both the arms, i.e., ARR ≤0.15, which corresponds to <1 relapse for every 6 years of patient exposure (**Figure 4B**).

Recently diagnosed, treatment-naive patients with high (compared to low) baseline sNfL levels experienced significantly more whole brain volume loss per year (relative difference: ofatumumab, 131.8%, p=0.007; teriflunomide, 146.6%, p<0.001; **Figure 4C**). Similar to the results in all RMS patients, newly diagnosed, treatment-naive patients with high sNfL (compared to low) levels at baseline lost more thalamus volume in the teriflunomide group (relative difference: 149.6%, p=0.003), with a similar but non-significant trend also noted in the ofatumumab group (relative difference: 174.5%, p=0.093; **Figure 4D**). In addition, high baseline sNfL (vs. low) was prognostic of a higher annual percentage volume change in WM in both treatment arms (relative difference: ofatumumab, 182.1%, p=0.022; teriflunomide, 776.3%, p=0.002; **Figure 4E**) but did not show a significantly prognostic value for change in cGM volume, although the trend was in the same direction (relative difference: ofatumumab, 143.8%, p=0.053; teriflunomide, 25.4%, p=0.408; **Figure 4F**). Similar to overall population, the differences in the time to first 3mCDW and 6mCDW were not statistically significant between high and low sNfL groups (data not shown) but demonstrated a consistent trend.

##### 3.2.2.2 Correlations Between sNfL and Regional Brain Volume Change

Similar to the results in the overall study population, of all the brain compartments analyzed, relative changes in volume were most pronounced in the thalamus. Baseline sNfL correlated with the percentage change from baseline to month 12 and month 24 in whole brain volume, WM volume, and thalamic volume, and, to a lesser degree, with cGM volume change. The strongest association of sNfL was with thalamic volume loss, both over 1 (ofatumumab, r=−0.379; teriflunomide, r=−0.399, both p<0.0001) and 2 years (−0.473; −0.460, both p<0.0001; **Table 2**; **Figures 3C, D**).

#### 3.2.3 Treatment Effect of Ofatumumab by Baseline NfL Category

**Figure 5:**
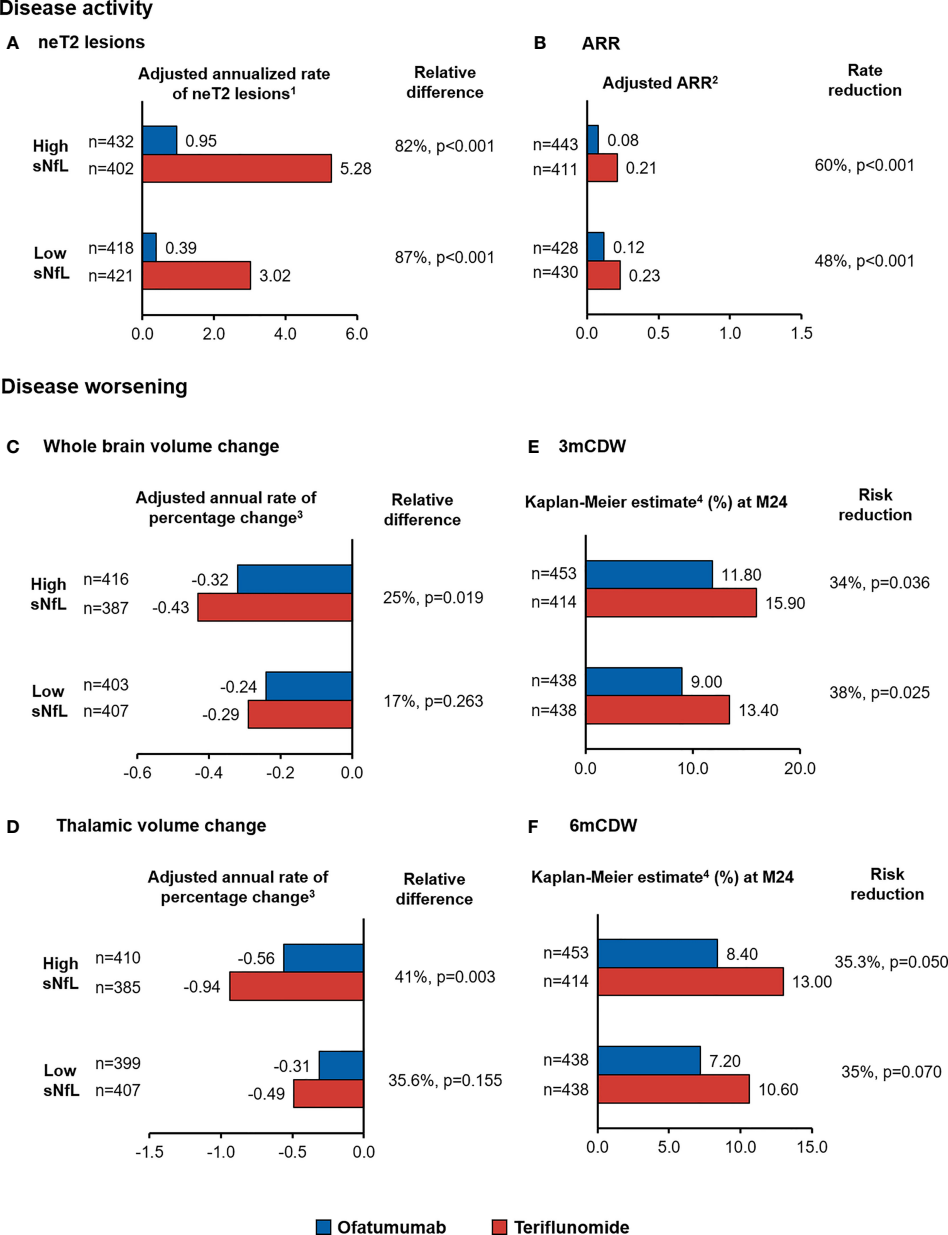
Treatment effect of ofatumumab compared with teriflunomide in the overall patient population, by baseline sNfL category, on measures of MS disease activity, namely, **(A)** neT2 lesions and **(B)** ARR, and disease worsening, namely, **(C)** whole brain volume change and **(D)** thalamic volume change time to **(E)** 3mCDW and **(F)** 6mCDW. ^1^Adjusted annualized mean rate of neT2 lesions. The number of neT2 lesions (compared to baseline or month 12) was analyzed in a negative binomial model with adjustments for treatment, baseline sNfL category, region and study as factors, and age, baseline volume of T2 lesions as continuous covariates, and treatment by baseline sNfL category interaction. The natural log of the time from the baseline scan (in years) was used as the offset. ^2^The ARR was analyzed in a negative binomial regression model with log-link to the number of relapses adjusted for treatment, baseline sNfL category, region and study as factors, number of relapses in previous year, baseline EDSS, baseline number of Gd+T1 lesions, and the patient’s age at baseline as covariates and treatment by baseline sNfL category interaction. The natural log of the time in study was used as offset to annualize the relapse rate. ^3^Adjusted annual rate of brain volume change as estimated in from the random coefficient model with study fit to the post-baseline assessments measured at M12, M24, and end-of-study, treatment as fixed effects (factors), and time as continuous covariates and treatment by time interaction for the overall analysis, with additional co-factors of subgroup, treatment by subgroup, and treatment by time by subgroup interactions for the subgroup analysis. Random terms for slopes and intercept are included. ^4^Cox regression adjusted study as stratum, treatment, region, and sNfL baseline high–low subgroup as factors and baseline EDSS as a continuous covariate along with treatment-by-sNfL baseline high-low subgroup interaction. ARR, annualized relapse rate; BVL, brain volume loss; 3mCDW, 3-month confirmed disability worsening; MRI, magnetic resonance imaging; neT2, new or enlarging T2 lesions; n.s., non-significant; sNfL, serum neurofilament light chain.

Ofatumumab significantly reduced T2 lesion formation compared with teriflunomide regardless of baseline sNfL with relative reductions of 82% and 87% in high and low sNfL groups, respectively (**Figure 5A**). ARR was significantly lower in the ofatumumab compared with teriflunomide arm (both p<0.001), corresponding to a relative reduction of 60% and 48% in the high and low sNfL groups, respectively (**Figure 5B**). In patients with high sNfL who were treated with ofatumumab, the rate of brain volume loss (between year 1 and 2) was lower than those treated with teriflunomide resulting in a relative reduction of 24.5% (−0.32 vs. −0.43, p=0.019) (**Figure 5C**). The treatment effect was consistent for reduction in thalamic volume loss with relative reduction of 40.6% (−0.56 vs. −0.94, p=0.003) (**Figure 5D**) but not statistically significant for WM or cGM volume loss. Greater effects of ofatumumab versus teriflunomide on reducing 3mCDW and 6mCDW risk were noted irrespective of baseline sNfL levels, corresponding to 34% and higher risk reduction in patients with high sNfL and low sNfL (**Figures 5E, F**).

## 4 Discussion

This protocol-planned analysis from the two large phase 3 ASCLEPIOS I and II trials confirms the prognostic value of baseline sNfL levels for on-study lesion formation and brain volume change in patients with RMS. The prognostic value of baseline sNfL on MRI lesion formation and brain volume loss over 1 to 2 years was consistently seen in both ofatumumab- and teriflunomide-treated patients, suggesting that the prognostic effect of sNfL is not limited to a specific treatment. Compared to patients with low sNfL levels in the same treatment group, patients with high sNfL levels at baseline developed 50%–150% more T2 lesions on-study, which is a relevant information to treating physicians, as it may influence long-term outcomes and, in combination with other assessments, guide treatment decisions. The prognostic value of baseline sNfL for lesion formation persisted in the second year of treatment in the teriflunomide arm, indicating that baseline sNfL has a clinically meaningful prognostic capacity beyond current assessments. Patients who were identified as having high NfL at baseline who were then randomized to teriflunomide had on average >5 new or enlarging T2 lesions in the first year of treatment, and lesion formation persisted to be high (>4 new or enlarging T2 lesions) in the second year of treatment. In contrast, high baseline NfL patients who were randomized to ofatumumab arm had 1.4 lesions in the first year of treatment but close to complete abrogation of lesion formation in the second year of treatment (0.09 lesions per year), showing that new demyelinating events could largely be prevented even in “high NfL” patients with highly efficacious treatment.

NfL assessments had added value over standard MRI assessments in our study. In patients who appeared free of Gd+ T1 lesions at baseline, and thus would be considered low risk patients based on the clinical and radiological assessment, it was shown that baseline sNfL could prognosticate the risk of future MS disease activity and worsening as measured by lesion formation and brain volume loss. This may be of relevance in the assessment of newly diagnosed or early MS patients in whom a reliable risk stratification is difficult in current practice. Two plausible mechanisms could explain the observed additional value of NfL over standard MRI in prognosticating future MS disease activity. First, MS-related spinal cord injury may be reflected in increased NfL levels, but it will not be invisible on a brain image. Second, NfL can be elevated for longer than gadolinium-enhancing lesions appearing visible on the radiological image, i.e., NfL may detect past lesion activity that cannot be detected anymore by a standard radiological assessment.

In our analysis, baseline sNfL correlated with whole brain and regional atrophy, with the strongest correlation with thalamic volume change. The thalamus is a small but particularly neuron-rich structure; it is a relay station for many long distance afferent and efferent white matter tracts and is affected early in MS (33). It has been previously shown that thalamic atrophy was associated with clinical disease progression (6, 7) and cognitive impairment (34) in MS. In a cross-sectional study including patients with MS, higher baseline sNfL levels were associated with lower baseline deep gray matter volumes, including thalamus, caudate, and hippocampus (35). More recent work, which focused on brain network hubs, revealed that a high T2 lesion volume at baseline was predictive of on-study cGM, deep GM, and thalamic volume loss (7). In both ASCLEPIOS studies, reduction in thalamic volume loss was slowed by ofatumumab compared with teriflunomide (ASCLEPIOS I: ofatumumab vs. teriflunomide, 1.00% vs. 1.40%, p=0.002; ASCLEPIOS II: −1.07 vs. 1.31%, p=0.044). These findings are in line with the disability outcomes of these phase 3 studies but different from results for whole brain volume change alone, which was not significantly different between treatment arms (30). Although this study was not powered to demonstrate a prognostic effect of sNfL on disability outcomes, as noted by the protocol, trends were directionally consistent with the results on whole brain and thalamus changes. It is of note that in these active controlled studies, approximately 90% of the ofatumumab-treated patients had no 3mCDW over up to 2.3 years, and in the teriflunomide arm, 85% of the patients remained free of 3mCDW, making it challenging to analyze disability outcomes in subgroups of patients. In clinical practice, one of the greatest challenges is the difficulty to prognosticate future disease trajectories and optimize treatment decisions at presentation because of the extreme variability of individual disease courses (14). A prognostic test, if standardized, could be a helpful tool in assessing the patient’s risk of future disease activity and worsening and could complement the clinical and radiological assessments. In this regard, periodic assessments of sNfL may help in assessing a patient’s risk of further subclinical disease activity and brain tissue loss and “silent progression” in RRMS patients (36). Especially early in the disease, much of the disease activity may be asymptomatic, and tissue damage is more difficult to recognize because of the high existing compensation and reorganization capacity (37). However, the radiological disease burden increases with each demyelinating event, and therefore, looking beyond the surface with a sNfL test may be particularly important. Our study conceptually confirms that sNfL has prognostic value for on-study lesion formation and brain volume loss also in the subgroup of recently diagnosed (within 3 years), treatment-naive patients. A high radiological disease burden (as measured by the T2 lesion volume) is a key factor that can predict further brain tissue loss (4), neuronal injury and putatively loss (3), and unfavorable long-term outcomes in patients (38). The prognostic value of baseline sNfL for lesion formation, brain volume loss, and directionally consistent results on disability worsening supports the clinical meaningfulness of the prognostic value of sNfL for future disease activity and worsening. In the active controlled ASCLEPIOS I and II studies, we could not confirm a prognostic effect on the ARR, which was low in both treatment arms (one relapse for every 9 years on ofatumumab versus one relapse for every 4 years in the teriflunomide arm). However, lesion formation and clinical relapses are both expressions of the same underlying pathophysiology, and it seems plausible that without active treatment, the prognostic effect on inflammatory demyelinating disease activity as demonstrated on lesion formation would have translated to a corresponding result in clinical relapses, as this was shown in previous studies with higher levels of disease activity (3, 9, 26, 29, 39, 40).

Irrespective of the sNfL level at baseline, in both ASCLEPIOS I and II studies treatment with ofatumumab significantly reduced sNfL concentrations relative to treatment with teriflunomide by 8.7% at the first assessment at month 3, 26.5% at month 12, and 23.8% at month 24 (p<0.001 for all time points) (30). A continuous reduction in sNfL levels as seen with ofatumumab treatment, corresponding to a reduction in irreversible neuronal loss, is likely a meaningful patient outcome.

As strengths of the present study, all analyses (including NfL thresholds, subgroup definitions, and statistical methodology) were pre-specified in the study protocol and the corresponding statistical analysis plan of ASCLEPIOS I and II, reducing the chances of reporting bias. For brevity, the results for the combined ASCLEPIOS I and II Phase 3 studies were reported. However, similar analyses were conducted in each study separately. Results of the two studies were independent as patients, centers, and investigators could only participate in one of the studies. Results of these two large studies in a total of 1,882 patients were highly consistent, individually confirming the prognostic value of baseline sNfL for on-study lesion formation and brain volume loss in both the ofatumumab and teriflunomide arms.

As a limitation and based on the pre-planned nature of the analysis, patients were stratified into “high” or “low” sNfL value at baseline with the intention to divide a typical RMS population (as defined by the inclusion and exclusion criteria of the ASCLEPIOS I and II studies) into groups of equal size with higher vs. lower than median sNfL. Recent work on sNfL threshold optimization suggests that the risk of disease activity and worsening increases gradually with increasing sNfL levels and that there is a relationship of increasing sNfL concentrations with older age, which becomes more prominent above the age of 50 years (41). It is likely that the threshold could be optimized further in future analyses and with a specific target endpoint in mind. Any such cut-point optimization should be done in the context of the intended use of an *in vitro* diagnostic device development to ensure the utility of such a tool in clinical practice. Our study demonstrated prognostic value of sNfL for on-study disease activity and worsening for ofatumumab and teriflunomide in the independent Phase 3 ASCLEPIOS I and II trials.

In conclusion, baseline sNfL levels are prognostic for on-study lesion formation and brain volume loss, in all RMS patients but specifically also in early treatment-naive patients. Irrespective of the baseline sNfL levels, ofatumumab consistently reduced sNfL concentrations relative to treatment with teriflunomide. The relations shown between baseline sNfL and both T2 lesion occurrence and brain volume loss during the study support that sNfL levels are a prognostic indicator of tissue damage. This study also supports the potential prognostic value of both sNfL and T2 lesions as predictors of higher probability of clinical progression in patients with MS.

## Data Availability Statement

The raw data supporting the conclusions of this article will be made available by the authors, without undue reservation.

## Ethics Statement

The studies involving human participants were reviewed and approved by relevant institutional review board or ethics committee at each trial site. The patients/participants provided their written informed consent to participate in this study.

## Author Contributions

TZ, DA, EA, AC, SH, LK, JK, HK, KR, MM, and WS contributed to study concept, data interpretation, and manuscript development. RW, BL, and PK contributed to data analysis and interpretation and manuscript development. DH contributed to study concept, data analysis and interpretation, and manuscript development. All authors contributed to the article and approved the submitted version.

## Conflict of Interest

TZ has received compensation for consulting and lecturing from Alexion, Biogen, Celgene, Novartis, Roche, Sanofi, and Teva and for research from Biogen, Novartis, Roche, Teva, and Sanofi.

DA reports consulting fees from Albert Charitable Trust, Alexion Pharma, Biogen, Celgene, Frequency Therapeutics, Genentech, Med-Ex Learning, Merck, Novartis, Population Council, Receptos, Roche, and Sanofi-Aventis, grants from Biogen, Immunotec and Novartis, and an equity interest in NeuroRx.

EA has received compensation for consulting from Actelion, Alexion, Biogen, Celgene, EMD Serono, Genentech, Novartis, Sanofi, and TG Therapeutics and for research from Biogen, Genentech, Novartis, TG therapeutics, Patient-Centered Outcomes Research Initiative, National Multiple Sclerosis Society, National Institutes of Health, and Rocky Mountain MS Center.

AC has received personal compensation for consulting or serving on scientific advisory boards from Biogen, Celgene, EMD Serono, Genentech/Roche, Greenwich Biosciences, Horizon, Janssen, Novartis, and TG Therapeutics, and for research from Genentech and EMD Serono.

SH has received personal compensation from Annexon, Alector, Bionure, and Neurona and has also received travel reimbursement from F. Hoffmann-La Roche and Novartis for CD20-related meetings and presentations.

LKs' institution (University Hospital Basel) has received the following exclusively for research support: Steering committee, advisory board and consultancy fees (Actelion, Bayer HealthCare, Biogen, BMS, Genzyme, Janssen, Merck, Novartis, Roche, Sanofi, Santhera, TG Therapeutics); speaker fees (Bayer HealthCare, Biogen, Merck, Novartis, Roche and Sanofi); support of educational activities (Allergan, Bayer HealthCare, Biogen, CSL Behring, Desitin, Genzyme, Merck, Novartis, Roche, Pfizer, Sanofi, Shire and Teva); license fees for Neurostatus products; and grants (Bayer HealthCare, Biogen, European Union, InnoSwiss, Merck, Novartis, Roche, Swiss MS Society and Swiss National Research Foundation).

JK’s institution (University Hospital Basel) has received and used exclusively for research support: consulting fees from Biogen, Novartis, Protagen AG, Roche, and Teva; speaker fees from the Swiss MS Society, Biogen, Novartis, Roche, and Genzyme; travel expenses from Merck Serono, Novartis, and Roche; and grants from the ECTRIMS Research Fellowship Programme, University of Basel, Swiss MS Society, Swiss National Research Foundation (320030_160221), Bayer AG, Biogen, Genzyme, Merck, Novartis, and Roche.

RW, BL, PK, HK, KR, MM, WS, and DH are employed by Novartis. This study was funded by Novartis Pharma AG, Basel, Switzerland. Novartis Pharma AG supported the development of this manuscript, provided data analyses according to the direction of the authors, and paid for medical writing support.

## Publisher’s Note

All claims expressed in this article are solely those of the authors and do not necessarily represent those of their affiliated organizations, or those of the publisher, the editors and the reviewers. Any product that may be evaluated in this article, or claim that may be made by its manufacturer, is not guaranteed or endorsed by the publisher.

## Supplementary Material

Supplementary Figure 1 Prognostic value of baseline sNfL category (high [red]: ≥9.3 pg/ml, low [green]: <9.3 pg/ml) on the annualized rate of neT2 lesion formation per year in patients **(A)** with Gd+ T1 lesions and **(B)** without Gd+ T1 lesions. Comparisons are between high vs low sNfL categories. ^1^Adjusted annualized mean rate of neT2 lesions. The number of neT2 lesions (compared to baseline) was analyzed in a negative binomial model with adjustments for treatment, baseline sNfL category, region and study as factors, and age, baseline volume of T2 lesions as continuous covariates, and treatment by baseline sNfL category interaction. The natural log of the time from the baseline scan (in years) was used as the offset. ^2^Indicates statistical significance (2-sided) at the 0.05 level. CI, confidence interval; Gd+, gadolinium-enhancing; neT2, new or enlarging T2 lesions; sNfL, serum neurofilament light.

Supplementary Figure 2 Prognostic value of baseline sNfL category (high [red]: ≥9.3 pg/ml, low [green]: <9.3 pg/ml) on the annual rate of whole brain volume change1 in the subgroup of patients **(A)** with Gd+ T1 lesions and **(B)** without Gd+ T1 lesions.Comparisons are between high vs low sNfL categories. ^1^Adjusted mean annual rate of percent change from baseline obtained from a random coefficients model with study, treatment, NfL high-low subgroup, and region as factors, time, number of Gd+ T1 lesions at baseline, baseline T2 volume, and normalized volume of the analyzed compartment at baseline as continuous covariate, and treatment-by-time-by-subgroup interaction as well as the 3 corresponding 2-way interactions. Random terms for slopes and intercept are included. ^2^Indicates statistical significance (2-sided) at the 0.05 level. BVL, brain volume loss; CI, confidence interval; cGM, cortical gray matter; Gd+, gadolinium-enhancing; LS, least square; sNfL, serum neurofilament light; WM, white matter.
