# Supplementary figures and images for: Prognostic Value of Serum Neurofilament Light Chain for Disease Activity and Worsening in Patients With Relapsing Multiple Sclerosis: Results From the Phase 3 ASCLEPIOS I and II Trials
Source: Front Immunol. 2022 Mar 31;13:852563. doi: 10.3389/fimmu.2022.852563 (PMC9009385; doi:10.3389/fimmu.2022.852563)

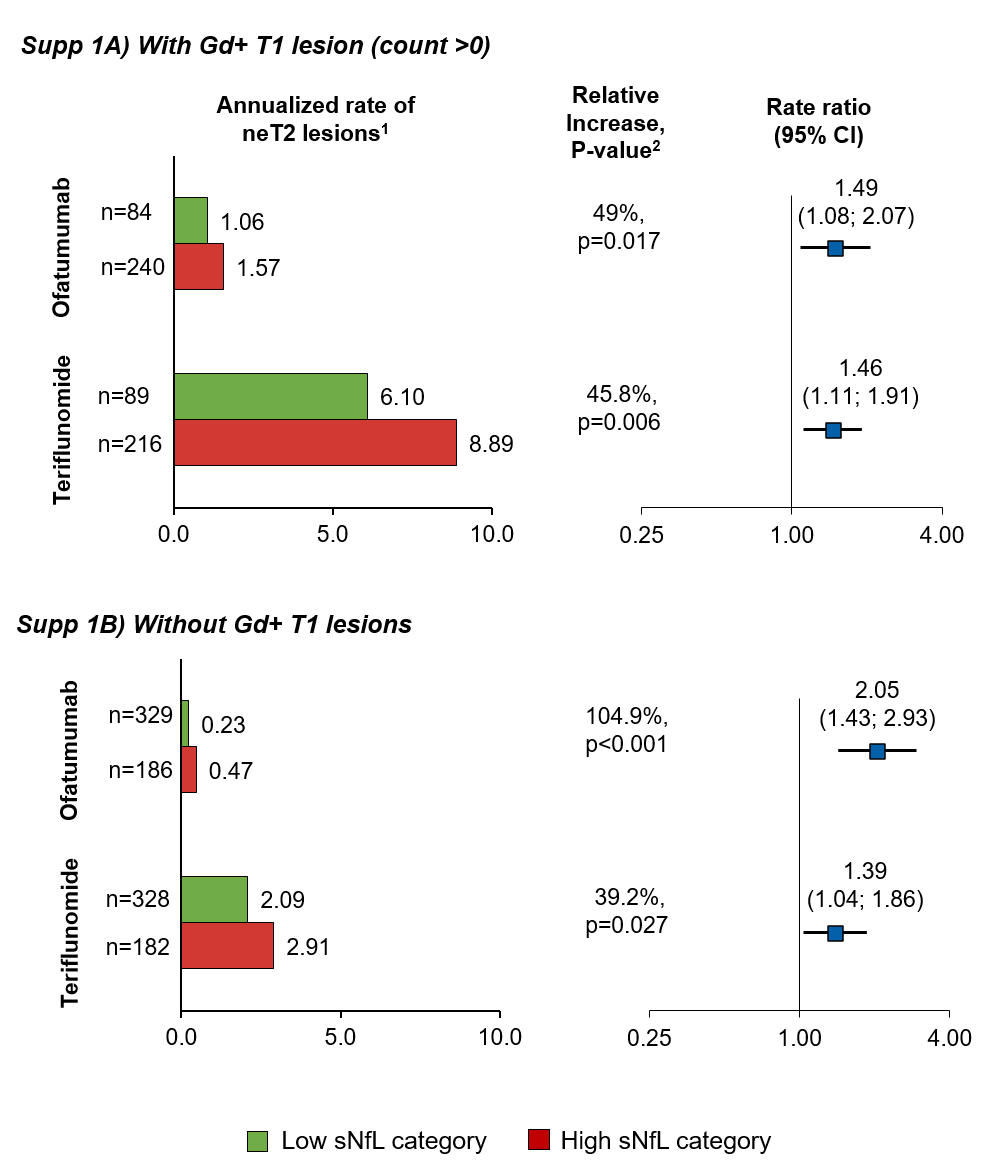

Supplement: Supplementary Figure 1 — Prognostic value of baseline sNfL category (high [red]: ≥9.3 pg/ml, low [green]: <9.3 pg/ml) on the annualized rate of neT2 lesion formation per year in patients (A) with Gd+ T1 lesions and (B) without Gd+ T1 lesions. Comparisons are between high vs low sNfL categories. 1Adjusted annualized mean rate of neT2 lesions. The number of neT2 lesions (compared to baseline) was analyzed in a negative binomial model with adjustments for treatment, baseline sNfL category, region and study as factors, and age, baseline volume of T2 lesions as continuous covariates, and treatment by baseline sNfL category interaction. The natural log of the time from the baseline scan (in years) was used as the offset. 2Indicates statistical significance (2-sided) at the 0.05 level. CI, confidence interval; Gd+, gadolinium-enhancing; neT2, new or enlarging T2 lesions; sNfL, serum neurofilament light. [file Image_1.tif]

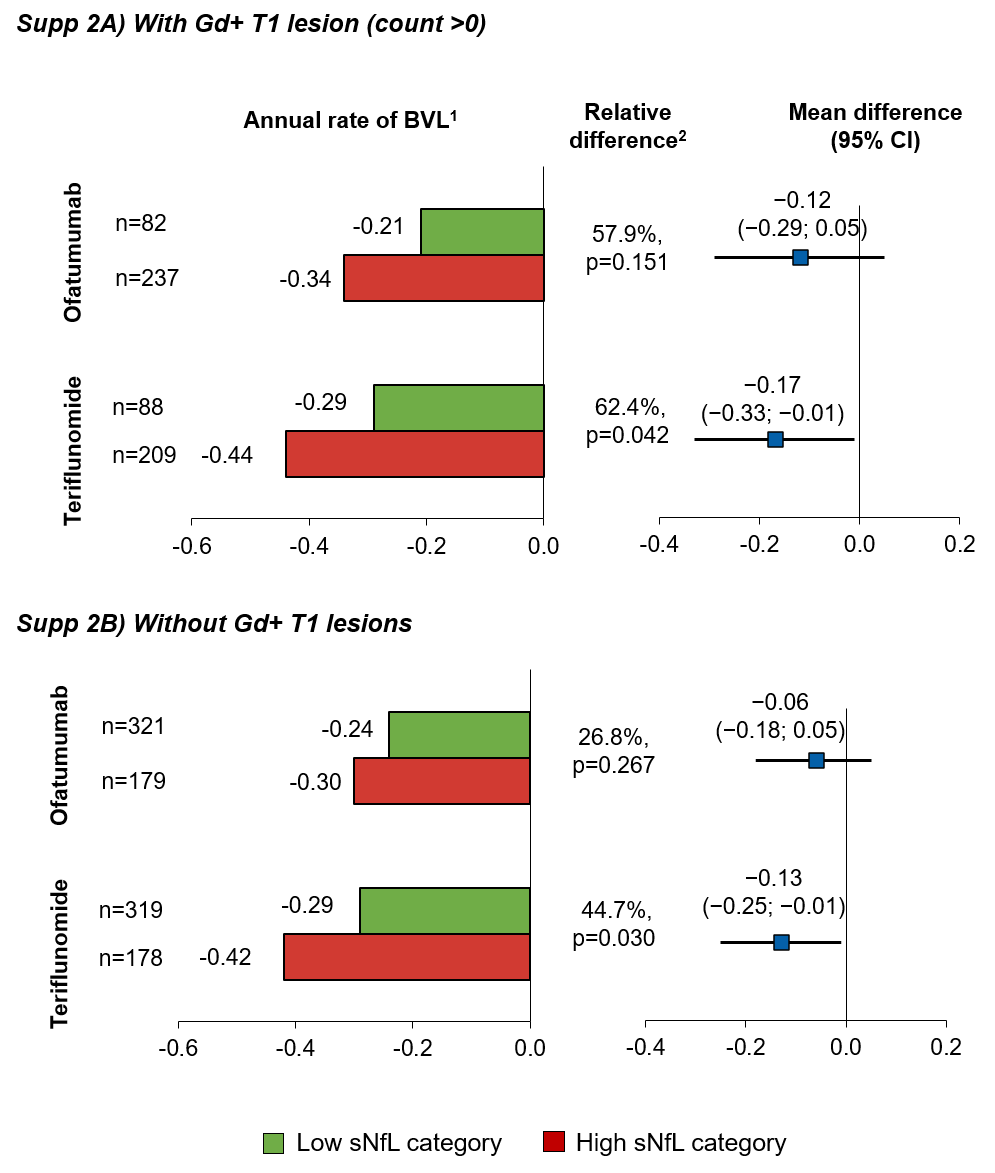

Supplement: Supplementary Figure 2 — Prognostic value of baseline sNfL category (high [red]: ≥9.3 pg/ml, low [green]: <9.3 pg/ml) on the annual rate of whole brain volume change1 in the subgroup of patients (A) with Gd+ T1 lesions and (B) without Gd+ T1 lesions.Comparisons are between high vs low sNfL categories. 1Adjusted mean annual rate of percent change from baseline obtained from a random coefficients model with study, treatment, NfL high-low subgroup, and region as factors, time, number of Gd+ T1 lesions at baseline, baseline T2 volume, and normalized volume of the analyzed compartment at baseline as continuous covariate, and treatment-by-time-by-subgroup interaction as well as the 3 corresponding 2-way interactions. Random terms for slopes and intercept are included. 2Indicates statistical significance (2-sided) at the 0.05 level. BVL, brain volume loss; CI, confidence interval; cGM, cortical gray matter; Gd+, gadolinium-enhancing; LS, least square; sNfL, serum neurofilament light; WM, white matter. [file Image_2.tif]
